# Homœopathy in New York City

**Published:** 1890-01

**Authors:** H. Hitchcock


### HOMŒOPATHY IN NEW YORK CITY.

Please take notice!

"The definition of a homœopathic physician is 'one who is a member of the Homœopathic County Medical Society.' No matter if I have recourse to allopathic remedies, I am a homœopathic physician as long as I am a member of this Society.

"Timothy F. Allen, M. D."

As reported in the *N. Y. Tribune,* Dec. 19th, '89.

This is the New York City standard!

No principles are necessary!

Homœopathy is but a sectarian name!

The Homœopathic Medical College and Hospital of the city of New York, of which Dr. Allen is the Dean, supports and upholds these doctrines, and this accounts for the fact that its graduates do not know anything about the principles, laws, and practice of Homœopathy.

The founder of Homœopathy proclaimed a law irrefutable, eternal, which should govern its practice. This has been thrown to the winds, because it compelled deep research and scientific procedures. Principles are of no value—popularity and power are everything. H. Hitchcock, M. D.

